# Quality of life and life satisfaction are severely impaired in patients with long-term invasive ventilation following ICU treatment and unsuccessful weaning

**DOI:** 10.1186/s13613-018-0384-8

**Published:** 2018-03-16

**Authors:** Sophie Emilia Huttmann, Friederike Sophie Magnet, Christian Karagiannidis, Jan Hendrik Storre, Wolfram Windisch

**Affiliations:** 1 Department of Pneumology, Cologne-Merheim Hospital, Kliniken der Stadt Köln gGmbH, Witten/Herdecke University Hospital, Ostmerheimer Strasse 200, 51109 Cologne, Germany; 2 Department of Pneumology, University Medical Hospital, Freiburg, Germany; 3 Department of Intensive Care, Sleep Medicine and Mechanical Ventilation, Asklepios Fachkliniken Munich-Gauting, Gauting, Germany

**Keywords:** Health-related quality of life, Home mechanical ventilation, ICU outcome, Respiratory failure, Tracheostomy, End of life

## Abstract

**Background:**

Health-related quality of life (HRQL), life satisfaction, living conditions, patients’ attitudes towards life and death, expectations, beliefs and unmet needs are all poorly understood aspects associated with patients receiving invasive home mechanical ventilation (HMV) following ICU treatment and unsuccessful weaning. Therefore, the present study aimed to assess (1) HRQL, (2) life satisfaction and (3) patients’ perspectives on life and death associated with invasive HMV as the consequence of unsuccessful weaning.

**Results:**

Patients undergoing invasive HMV with full technical supply and maximal patient care were screened over a 1-year period and assessed in their home environment. The study comprised the following: (1) detailed information on specific aspects of daily life, (2) self-evaluation of 23 specific daily life aspects, (3) HRQL assessment using the Severe Respiratory Insufficiency Questionnaire, (4) open interviews about the patient’s living situation, HRQL, unsolved problems, treatment options, dying and the concept of an afterlife. Out of 112 patients admitted to a specialized weaning centre, 50 were discharged with invasive HMV and 25 out of these (14 COPD and 11 neuromuscular patients) were ultimately enrolled. HRQL and life satisfaction were severely impaired, despite maximal patient care and full supply of technical aids. The most important areas of dissatisfaction identified were mobility, communication, social contact and care dependency. Importantly, 32% of patients would have elected to die in hindsight rather than receive invasive HMV.

**Conclusions:**

Despite maximal patient care and a full supply of technical aids, both HRQL and life satisfaction are severely impaired in many invasive HMV patients who have failed prolonged weaning. These findings raise ethical concerns about the use of long-term invasive HMV following unsuccessful weaning.

**Electronic supplementary material:**

The online version of this article (10.1186/s13613-018-0384-8) contains supplementary material, which is available to authorized users.

## Background

Long-term home mechanical ventilation (HMV) is an increasingly used treatment option for patients with chronic respiratory failure [1, 2]. For this purpose, HMV can be performed either invasively following tracheotomy, or noninvasively using face masks, the latter being the preferred mode [1]. Invasive HMV is only chosen in cases where noninvasive HMV is no longer feasible or sufficient [3]. Here, particularly in patients with neuromuscular disorders (NMDs), invasive HMV should only be electively established after detailed, fully informed consent is given for the procedures involved and their potential consequences [3].

In addition, intubation of ICU patients suffering from acute respiratory failure is often accompanied by tracheotomy if mechanical ventilation (MV) has to be applied for a longer period, or if there are foreseeable difficulties with weaning [4]. Even though many patients can eventually be liberated from invasive MV once the acute respiratory failure has been successfully treated, some still require prolonged weaning [4]. In the event that this fails, invasive HMV must once again be implemented [5].

Such patients do not usually have the opportunity during stable phases of their disease to decide whether or not they wish to become tracheotomized. While there is increasing evidence that outcome and health-related quality of life (HRQL) are improved in many patients receiving noninvasive HMV [2, 6], the impact of invasive HMV remains especially unclear in patients receiving invasive HMV after an unsuccessful attempt at weaning.

According to the Severe Respiratory Insufficiency Questionnaire (SRI), a specific HRQL measuring tool [6–8] (https://www.pneumologie.de/service/patienteninformation/patienten-fragebogen-zur-befindlichkeit-bei-schwerer-respiratorischer-insuffizienz/), we found that HRQL differed substantially among patients undergoing invasive HMV primarily following weaning failure, with scores ranging from very good to very poor [9]. Older patients with chronic obstructive pulmonary disease (COPD) and more co-morbidities showed a higher tendency for reduced HRQL than patients with NMD. In addition, some patients verbally expressed the severe limitations they faced in daily life [9]. Therefore, it appears that even the most specific questionnaires cannot fully assess the specific and complex living conditions of patients with invasive HMV.

The present study therefore aimed to assess (1) HRQL, (2) life satisfaction and (3) patients’ perspectives on life and death associated with invasive HMV following unsuccessful weaning in patients with intubation and subsequent tracheostomy that have become necessary to treat acute-on-chronic respiratory failure by carrying out detailed assessments of the specific living conditions experienced by patients in their respective home environments. The purpose of this was to identify specific problems and undiscovered needs of these patients, as well as the reasons for reduced HRQL and life satisfaction following a new study different from the authors’ previous one [9].

## Methods

The study protocol was approved by the local ethics committee (Ethikkommission der Ärztekammer Nordrhein, Germany) and performed in accordance with the ethical standards laid down in the Declaration of Helsinki. The study was registered under the German Clinical Trials Register (DRKS00006524) with the Universal Trial Number (UTN): U1111-1159-5354. Informed written consent was obtained from all subjects or legal guardians.

### Subjects

The study was performed in adult tracheotomized patients undergoing long-term invasive HMV. All patients were treated on a specialized weaning unit for prolonged weaning (Department of Pneumology, Cologne-Merheim Hospital, University Witten/Herdecke, Germany) following the need for intubation and tracheotomy due to acute respiratory failure prior to the study. Specifically, the weaning unit was accredited by the German Society of Pneumology and Mechanical Ventilation (DGP) and aims for tracheotomized patients who are ready to wean, but who still fit with the category of prolonged weaning according to international criteria while decannulation has not become successful in the external referring hospital [5].

For the purpose of the study, we screened all patients who underwent prolonged weaning (as defined by international and national guidelines [5, 10]) and were treated on the specialized weaning unit between January and December 2014. Eligible patients who died during the recruitment period, as well as those who were successfully weaned from invasive ventilation, with or without the adjunct of NIV, were not included in the final study. Thus, only patients who were discharged to an outpatient environment to continue invasive HMV following unsuccessful weaning were included in the study. A prerequisite for the study was that patients had to be acclimatized to their home environment; therefore, only patients who underwent invasive HMV for at least 2 months were included. Since the patient’s ability to perceive his or her own situation was mandatory for the study, severe mental retardation served as an exclusion criterion.

### Study design

All patients were visited in their home environment by a physician experienced with ICU medicine and prolonged weaning (first author) who was also experienced in performing interviews in these patients in the home environment according to previous research [9]. Four consecutive steps were performed for each patient:

1. Detailed information about sociodemographics, medical history, living situation, nursing care, medical care, MV, supply of technical aids, treatments (physiotherapy, occupational therapy and speech therapy), nursing dependency and daily living activities, social contacts, daily routine, legal guardianship, and religion/faith was collected. Information from medical documents was also recorded, and inspection of the home environment as well as interviews with patients, caregivers and relatives was carried out.
2. Based on the collated information, 23 important aspects of living with invasive HMV were defined following discussion and final agreement within the expert panel (all authors). Here, patients were required to indicate whether or not they were satisfied with each of these conditions (yes/no). Care was taken to ensure that the patients’ opinion was exclusively assessed, whereby relatives, caregivers or other people were not allowed to answer these questions.
3. Patients completed the original German version of the SRI, an instrument specifically designed to measure HRQL in patients with severe respiratory insufficiency [6–8]. The SRI Questionnaire contains 49 items with seven subscales measuring different aspects of HRQL (Respiratory Complaints, Physical Functioning, Attendant Symptoms and Sleep, Social Relationships, Anxiety, Psychological Well-being, Social Functioning). Each subscale produces a score (0–100), with lower scores indicating poorer health status. The scales can be aggregated to one Summary Score. Answers are given on a 5-point Likert scale ranging from “completely untrue” to “always true”. Again, relatives and caregivers were excluded from answering questions.
4. An open interview was performed by posing questions about (1) the living situation, (2) quality of life, (3) unsolved problems regarding the underlying disease, (4) treatment options, (5) dying, and (6) the concept of an afterlife. Again, the six topics for the open interview were determined within the expert group.

### Statistical analysis

Demographics, numeric data and SRI values were subjected to normality testing using the Shapiro–Wilk test. All normally distributed data are presented as mean ± standard deviation. Non-normally distributed data (Shapiro–Wilk with *P* value < 0.05) are provided as median values with minimum and maximum values. Binary data are presented with absolute numbers and percentages [*n* (%)].

Group comparison of SRI results was performed with respect to (1) the underlying disease (NMD vs. COPD) and (2) the individual’s attitude towards tracheostomy and invasive HMV (no regret vs. regret). Therefore, paired *t* tests were used for normally distributed data. A nonparametric test (Wilcoxon–Mann–Whitney rank-sum test) was used on non-normally distributed data. Group effects were estimated with 95% confidence intervals and tested with a 2-sided level of 0.05.

## Results

**Fig. 1:**
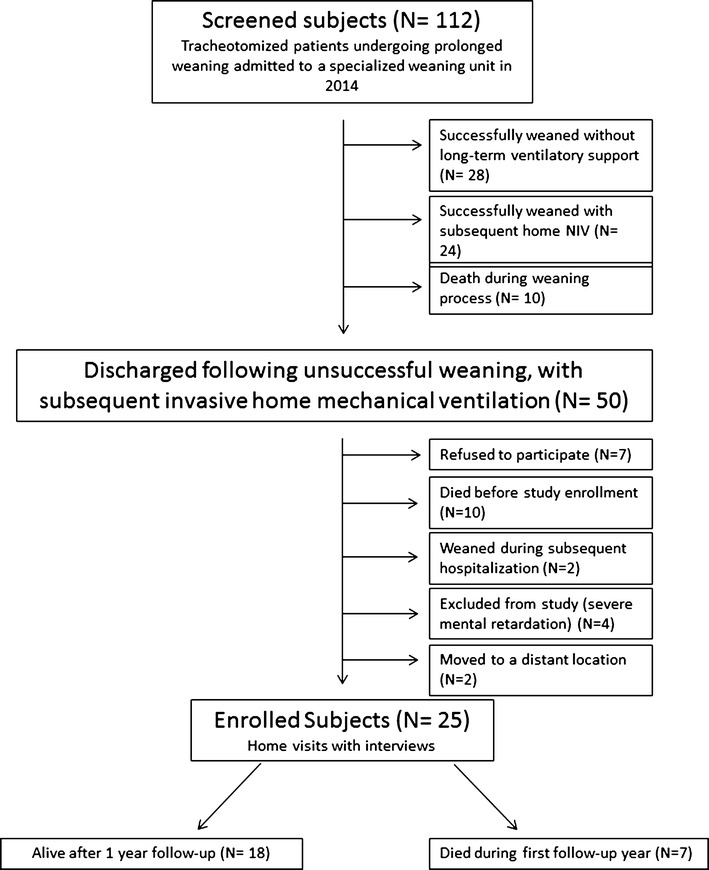
Flow chart of the study. *NIV* noninvasive ventilation

A flow chart of the study cohort is displayed in Fig. 1.

**Fig. 2:**
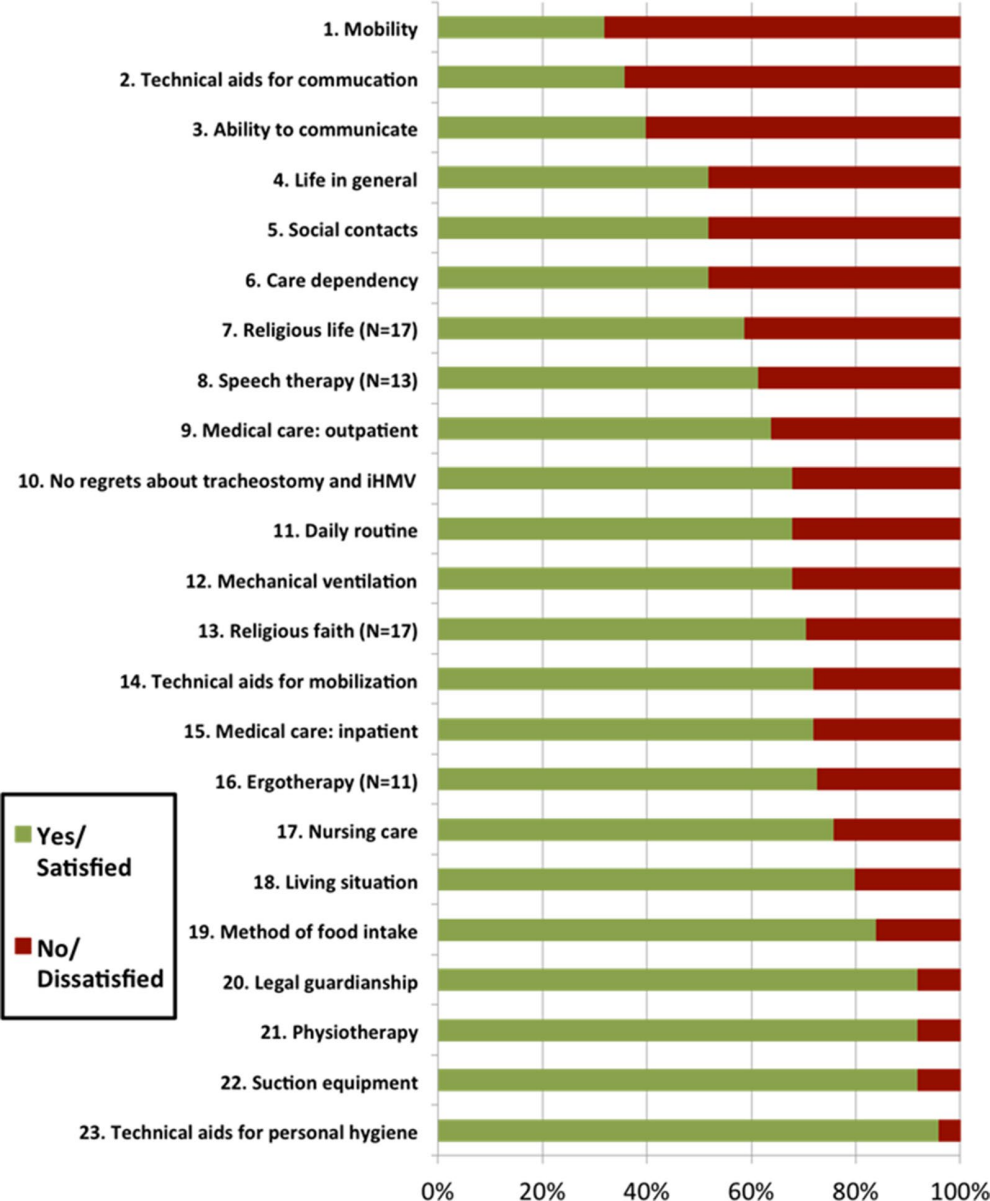
Subjective evaluation of life satisfaction in relation to 23 specific aspects of life in ascending order. *iHMV* invasive home mechanical ventilation

A total of 25 patients (10 females, median age 64 years, min/max 20;82 years, 14 primarily with COPD, 11 with NMD) were visited in their home environment and intensively studied as outlined in Methods section. Further demographic data, disease classifications, co-morbidities, but also patients’ marital statuses and education are listed in Additional file 1: Tables S1 and S2, respectively. Self-evaluation of the relevant daily life aspects is illustrated in Fig. 2.

Overall, 23 different topics were rated by the patients. Data are also provided according to disease categories, showing that COPD patients are more frequently unsatisfied than neuromuscular patients with regard to the 23 listed aspects (Additional file 1: Table S3). Adding to this, detailed information on the underlying circumstances for each of the 23 topics is listed in Additional file 1: Table S4.

**Fig. 3:**
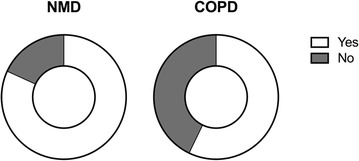
Degree of satisfaction with history of tracheostomy according to underlying disease—question: In hindsight, would you choose tracheostomy for long-term invasive HMV again? *NMD* neuromuscular disease, *COPD* chronic obstructive pulmonary disease, *HMW* home mechanical ventilation

**Fig. 4:**
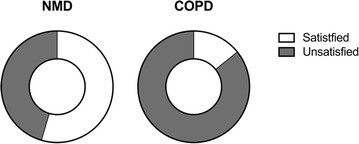
Degree of satisfaction with mobilization according to underlying disease—question: Are you satisfied with your level of mobility? *NMD* neuromuscular disease, *COPD* chronic obstructive pulmonary disease

As an example, two of the 23 topics (No. 10, Fig. 3 and No. 1, Fig. 4) are illustrated according to the underlying disease.

To this end, the topic most frequently reported as being unsatisfactory was mobility. In particular, a much larger proportion of COPD patients (85.7%) were unsatisfied with their mobility compared to NMD patients (45.5%) (Additional file 1: Table S3). Only one patient (4%) was able to get out of bed without help, 23 patients (92%) were dependent on technical aids and/or personal help and one patient (4%) could not leave the bed at all. However, all patients had individually prescribed technical aids for mobility such as wheel chairs, rollators and lifters (Additional file 1: Table S4). Furthermore, leaving the house with or without help was only possible for 16 patients (64%). Excursions and travelling were possible for 13 (52%) and two patients (8%), respectively.

Regarding the question about “choosing tracheostomy again, in hindsight”, 42.9% of COPD patients (*N* = 6) and 18.2% of NMD patients (*N* = 2; one with amyotrophic lateral sclerosis, one with spinal cord injury) indicated that they would have refused to have a tracheotomy if they had to choose again. Importantly, this question was raised under the assumption that the alternative to tracheotomy was death, as communicated to all patients during the interview. Unfortunately, it remains unclear whether some of these patients eventually asked for withdrawing of mechanical ventilation. Of note, tracheotomy dated back to a median of 23 months (min 6; max 145 months), with no difference between patients who refused and those who didn’t. Seven out of eight and 8/8 patients who would have refused a tracheotomy indicated dissatisfaction with MV and mobility, respectively. Finally, 18 patients (72%) had had unplanned hospital admissions for the management of acute deteriorations prior to the study.

Another important issue addressed in the interview was communication. In order to communicate, 21 (84%) patients required technical aids (Additional file 1: Table S4). Despite this, the ability to speak was impaired in 48% of patients, to write by hand in 24%, and to write using a computer in 48%. Remarkably, three NMD patients could only communicate with eye movements.

Most of the patients had family members and/or close friends (Additional file 1: Table S4). Fifteen patients (60%) lived with family members. In contrast, after invasive HMV was established, six (24%) and 14 patients (56%) lost contact with close family members and close friends, respectively. Patients were also highly dependent on nursing care: bathing (100%), dressing (96%), use of the toilet (92%), grooming (76%), feeding (44%).

Regarding outpatient care, 23 patients (92%) received home visits from a general practitioner and four patients (16%) were visited by a specialized respiratory physician. Nevertheless, all patients were assigned to a specialized ventilation centre with a median (min/max) distance of 15 km (0.1/104 km). Outpatient nursing care was provided in 92% of patients. In addition, family members were involved in the nursing care of 48% of patients: primarily in basic care and to a lesser extent in respiratory care. Eighteen patients (72%) lived in a private home and seven patients (28%) in a nursing facility (Additional file 1: Table S4). Nutrition was provided via percutaneous endoscopic gastrostomy in nine patients (36%).

The daily routine of the 25 patients who underwent invasive HMV is illustrated in Additional file 1: Figure S1 and Table S4. Importantly, patients spent most of their time watching television when they were not asleep (8 h per day). Eleven patients (44%) received continuous ventilation for 24 h per day. In contrast, 14 patients (66%) were intermittently able to breathe spontaneously, with a mean spontaneous breathing period of 8.5 ± 5.7 h per day.

 Information on the Subscales and Summary Scale of the SRI is provided in Table 1, with emphasis on the differences between the two patient groups, as well as between those who regretted their tracheostomy and those who didn’t. To this end, patients who regretted their tracheostomy had significantly lower SRI results for both the Subscale Anxiety and the Summary Scale, while most of the remaining scales also tended to be lower.

**Table 1:** Sub- and summary scales of the SRI according to underlying disease (NMD vs. COPD) and individual attitudes towards tracheostomy and invasive HMV

*N* = 25	Underlying disease			Attitude towards tracheostomy and invasive HMV		
	NMD (*N* = 11)	COPD (*N* = 14)	*P* value	No regrets (*N* = 17)	Regrets (*N* = 8)	*P* value
Respiratory complaints	52 ± 27	54 ± 27	0.912	56.7 ± 24.9	36.3 ± 21.5	0.059
Physical functioning	27 ± 23	13 (0;58)	0.267	16.7 (0;58,3)	22.9 ± 22.8	0.617
Attendant symptoms and sleep	58 ± 18	66 ± 26	0.393	63.0 ± 21.7	45.1 ± 26.9	0.087
Social relationships	64 ± 19	54 ± 27	0.278	63.9 ± 23.8	47.8 ± 14.5	0.136
Anxiety	58 ± 23	53 ± 35	0.693	58.2 ± 30.3	34.4 ± 24.0	0.063
Psychological well-being	51 ± 26	46 ± 27	0.267	*54.9* ± *21.0*	*34.0* ± *24.6*	*0.038*
Social functioning	33 ± 20	42 ± 23	0.320	40.9 ± 24.8	31.0 ± 22.3	0.350
Summary scale	49 ± 16	47 ± 20	0.814	*51.2* ± *18.3*	*35.9* ± *13.3*	*0.046*

Data are presented as mean ± standard deviation. For non-normally distributed data, median values with minimum and maximum ranges are given

*NMD* neuromuscular disorders; *HMV* home mechanical ventilation; *SRI* Severe Respiratory Insufficiency Questionnaire

The results of the open interview on the topics of life and death and afterlife are summarized in Table 2. Since the interview was too exhausting for one patient suffering from both COPD and obesity hypoventilation syndrome, detailed interviews are available for 24 patients. With regard to these results, 21 patients (84%) had a religious affiliation: Catholic (*N* = 13), Protestant (*N* = 5), Muslim (*N* = 2) and Hindu (*N* = 1). However, only 17 of these patients (68%) reported having an active faith.

**Table 2:** Summary of open interview (*N* = 24)

Questions 1–6
*1. How is your current living situation?* Three patients said that they are able to cope with their respective living conditions Twenty-one patients felt lousy, stressed and massively impaired. Emotions such as anxiety and sadness, and feelings of being dependent and waiting for death were frequently reported
*2. How would you assess your quality of life? What makes your life worth living?* Seventeen patients emphasized that despite their reduced quality of life, the deep relationship with family members and (to a lesser extent) friends, nevertheless, made life worth living. Among these patients, two had the hope of eventually becoming weaned and healed, respectively Seven patients reported a severely reduced quality of life with nothing available to make it worth living again
*3. What are your wishes regarding the treatment of your disease. What are the unresolved problems?* Fourteen patients had wishes that related to their treatment: better ability to speak (*N* = 1), less pain (*N* = 2), no further disease progression (*N* = 1), lung transplantation (*N* = 1), definitive weaning (*N* = 2), more awareness (*N* = 2), technical advances aimed at healing (*N* = 5) Ten patients had no wishes relating to their treatment
*4. What are your wishes regarding your ventilation therapy? How could this potentially be improved?* Fourteen patients had wishes that related to potential improvements in ventilation therapy: Switching to NIV (*N* = 1), less dyspnoea (*N* = 1), no further admissions to hospital (*N* = 2), longer periods of spontaneous breathing (*N* = 5), definitive weaning (*N* = 5) Ten patients had no wishes relating to potential improvements in ventilation therapy
*5. What do you think about dying and death?* Seventeen patients did think about dying and their own death: two patients expressed the wish to die, seven patients had a fear of dying/suffering during dying, and eight patients had no fear of dying Seven patients thought neither about dying, nor their own death
*6. Do you believe in an afterlife? What do you think is going to happen after you die?* Eleven patients believed in an afterlife: two patients had no idea how life after death would be, and nine patients had hopes (being with family members, being free, an eternal life, transmigration of souls, resurrection, being a spirit) Thirteen patients did not believe in an afterlife

## Discussion

This is the first study to provide a detailed description of patient characteristics, living conditions, specific aspects of HRQL and attitudes towards life, dying and death in patients with long-term invasive HMV following ICU treatment and unsuccessful weaning. The main finding was that HRQL and life satisfaction are massively impaired in many of these patients, despite maximal patient care and full supply of technical aids. Importantly, one-third of patients indicated that they would not have chosen to have a tracheostomy inserted if they had the chance to decide again, with this decision being based on the understanding that refusing a tracheostomy would have led to death. Importantly, based on the SRI scores and interview findings, these patients had the worst HRQL.

Overall, these findings raise ethical concerns about the use of long-term invasive HMV following unsuccessful weaning. In addition, the prognosis of patients with prolonged weaning in the present study was severely impaired. Some patients had already died during the weaning process, others died shortly after they were assigned to invasive HMV prior to study inclusion, and some died in the year following study inclusion. This is in line with recent research that also reported severely impaired prognoses of patients who failed the weaning process [11–13]. In addition, there is increasing evidence to suggest that weaning success rates and patient outcome steadily deteriorate over time, an effect attributed to the observation that more severely ill patients are admitted to weaning centres [12]. This is presumably due to the fact that greater numbers of chronically critical patients are surviving catastrophic illnesses as a result of modern ICU medicine [12]. However, despite the increased success of modern medicine in saving lives, the flipside is that surviving ICU treatment—but remaining dependent on invasive ventilation—is associated with a high risk of extremely poor quality of life. Moreover, there are reportedly many problems and individually raised discomforts and dissatisfactions voiced by HMV patients in their final months of life [14]. In addition, it has been emphasized that we need to increase our level of consideration for how patients with HMV die [15]. Therefore, ICU medicine should not simply focus on how to best preserve life; it should also consider the long-term living conditions of patients who remain dependent on long-term invasive ventilation. Finally, it remains unclear how many patients regretting getting tracheotomized would eventually asked for withdrawing of mechanical ventilation and how this can be brought to the clinician’s attention.

The outcome of the present study is somewhat in contrast to the findings reported by Marchese et al. [13], whereby 90% of patients undergoing invasive HMV would have chosen a tracheostomy again. However, the tracheotomies in the latter study were performed electively, and the patients were younger and had a promising median survival of 49 months. Furthermore, they were cared for primarily by family members, in contrast to our study population, whose primary care was provided by specialized nurses. In addition, the proportion of patients with pulmonary diseases was considerably lower in the Marchese study than the present one [13]. Of note, HRQL and life satisfaction in the present trial were particularly impaired in patients with COPD, in line with the previous observations [9]. Finally, patients in the current study had significant co-morbidities. Therefore, in the light of the evidence presented here and elsewhere, it is most likely that COPD patients in whom weaning has failed after ICU treatment may no longer have a life worth living.

The strength of the present study is that the comprehensive, detailed interview process that took place in the home environment provided meticulous details on how HMV patients live, feel and think. Of note, the two conditions that were associated with the most dissatisfaction were impaired mobility and communication. To this end, 36% of patients could not leave the house and 48% were unable to speak (Additional file 1: Table S3). This is remarkable, especially since aids for mobility and communication were thoroughly provided. Thus, in most cases it is the nature of the maximally advanced disease state that impairs life satisfaction, and this cannot be fully compensated by technical aids and patient care.

Social contact and care dependency were two additional aspects with which patients were dissatisfied. Importantly, patients spent most of their time watching television (median 8 h) during waking hours, with only a median social contact time of 1.5 h. In addition, many patients had lost contact with family members and close friends after the establishment of invasive HMV. However, 71% of the patients emphasized that they had a meaningful relationship with family members, and most were satisfied with their living conditions. To this end, 72% of patients lived in their own private home, and 60% of all patients lived with family members. Based on this finding, it is remarkable that nearly 50% of patients were not satisfied with their social situation. Finally, all patients were extremely dependent on nursing care, and the family members of 48% of patients were involved in patient care, indicating the close interlink between living situation, family contact and patient care. Of note, while there is no information available about how family members would evaluate their level of life satisfaction if a close relative become ventilator dependent in the home environment, previous research has indicated that family members were less satisfied with a relative having a tracheostomy than the patient him/herself [13].

Finally, there was a broad heterogeneity among patients regarding religious life, faith and belief in an afterlife. This was also dependent on different religious affiliations. Some patients had detailed thoughts about their deaths, ranging between positive and negative, and some even verbally expressed their wish to die. In contrast, other patients had no definitive thoughts on this subject and avoided thinking about death. The impact of religious life on HRQL and life satisfaction, however, needs to be further elucidated in future.

As a limitation of the current study and as the prize for the individually detailed investigation, the number of patients was low. In addition, this was a monocentric study. Therefore, it cannot be excluded that patients may respond differently under different conditions, particularly in other countries. Therefore, further studies in different countries are needed to verify the current findings.

## Conclusions

In conclusion, despite maximal patient care and a full supply of technical aids, both HRQL and life satisfaction are severely impaired in many invasive HMV patients who have failed prolonged weaning. The most important areas of dissatisfaction are mobility, communication, social contacts and care dependency. Importantly, one-third of patients would have preferred to die rather than receive invasive HMV. This raises ethical concerns about the practice of long-term MV following unsuccessful weaning, even though it still should be taken into account that some patients clearly benefit from long-term invasive HMV. Therefore, to avoid unethical prolongation of life, the disciplines of ICU medicine, prolonged weaning care and long-term outpatient care need to move closer together in order to improve individual decision-making processes that incorporate patients’ beliefs, expectations and circumstances.

## Additional file

**Additional file 1. Table S1:** Demographics, disease categories and co-morbidities. **Table S2:** Marital status and education. **Table S3:** Proportion of patients dissatisfied with specific aspects of daily life: NMD versus COPD. **Table S4:** Information on living conditions (N = 25). **Figure S1:** Daily routine of patients receiving invasive home mechanical ventilation.

## Abbreviations

COPD: chronic obstructive pulmonary disease
HMV: home mechanical ventilation
iHMV: invasive home mechanical ventilation
MV: mechanical ventilation
HRQL: health-related quality of life
NMD: neuromuscular disorders
SRI: Severe Respiratory Insufficiency Questionnaire

## References

[1] Lloyd-Owen SJ, Donaldson GC, Ambrosino N, Escarabill J, Farre R, Fauroux B, Robert D, Schoenhofer B, Simonds AK, Wedzicha JA (2005). Patterns of home mechanical ventilation use in Europe: results from the Eurovent survey. Eur Respir J.

[2] Windisch W. Home mechanical ventilation. In: Tobin MJ, editor. Principles and practice of mechanical ventilation, 3rd edn. p. 683–697. Nex Yoek: Mc Graw Hill Medical; 2012.

[3] Windisch W, Walterspacher S, Siemon K, Geiseler J, Sitter H (2010). Guidelines for non-invasive and invasive mechanical ventilation for treatment of chronic respiratory failure. Published by the German Society for Pneumology (DGP). Pneumologie.

[4] Beduneau G, Pham T, Schortgen F, Piquilloud L, Zogheib E, Jonas M, Grelon F, Runge I, Nicolas T, Grange S (2017). Epidemiology of weaning outcome according to a new definition. The WIND study. Am J Respir Crit Care Med.

[5] Boles JM, Bion J, Connors A, Herridge M, Marsh B, Melot C, Pearl R, Silverman H, Stanchina M, Vieillard-Baron A (2007). Weaning from mechanical ventilation. Eur Respir J.

[6] Windisch W (2008). Quality of life in home mechanical ventilation study g: impact of home mechanical ventilation on health-related quality of life. Eur Respir J.

[7] Windisch W, Freidel K, Schucher B, Baumann H, Wiebel M, Matthys H, Petermann F (2003). The Severe Respiratory Insufficiency (SRI) Questionnaire: a specific measure of health-related quality of life in patients receiving home mechanical ventilation. J Clin Epidemiol.

[8] Windisch W, Budweiser S, Heinemann F, Pfeifer M, Rzehak P (2008). The Severe Respiratory Insufficiency Questionnaire was valid for COPD patients with severe chronic respiratory failure. J Clin Epidemiol.

[9] Huttmann SE, Windisch W, Storre JH (2015). Invasive home mechanical ventilation: living conditions and health-related quality of life. Respiration.

[10] Schönhofer B, Geiseler J, Dellweg D, Moerer O, Barchfeld T, Fuchs H, Karg O, Rosseau S, Sitter H, Weber-Carstens S (2015). S2k-guideline “prolonged weaning”. Pneumologie.

[11] Schonhofer B, Euteneuer S, Nava S, Suchi S, Kohler D (2002). Survival of mechanically ventilated patients admitted to a specialised weaning centre. Intensive Care Med.

[12] Polverino E, Nava S, Ferrer M, Ceriana P, Clini E, Spada E, Zanotti E, Trianni L, Barbano L, Fracchia C (2010). Patients’ characterization, hospital course and clinical outcomes in five Italian respiratory intensive care units. Intensive Care Med.

[13] Marchese S, Lo Coco D, Lo Coco A (2008). Outcome and attitudes toward home tracheostomy ventilation of consecutive patients: a 10-year experience. Respir Med.

[14] Vitacca M, Grassi M, Barbano L, Galavotti G, Sturani C, Vianello A, Zanotti E, Ballerin L, Potena A, Scala R (2010). Last 3 months of life in home-ventilated patients: the family perception. Eur Respir J.

[15] Windisch W (2010). Home mechanical ventilation: who cares about how patients die?. Eur Respir J.

