# Supplementary material for: Quality of life and life satisfaction are severely impaired in patients with long-term invasive ventilation following ICU treatment and unsuccessful weaning
Source: Ann Intensive Care. 2018 Mar 16;8:38. doi: 10.1186/s13613-018-0384-8 (PMC5856853; doi:10.1186/s13613-018-0384-8)

**Quality of life and life satisfaction are severely impaired in patients with long-term invasive ventilation following ICU treatment and unsuccessful weaning**

Sophie Emilia Huttmann^1^, Friederike Sophie Magnet^1^, Christian Karagiannidis^1^, Jan Hendrik Storre^2,3^, Wolfram Windisch^1^

# ^1^Department of Pneumology, Cologne-Merheim Hospital, Kliniken der Stadt Koeln, Witten/Herdecke University Hospital, Cologne, Germany

# ^2^Department of Pneumology, University Medical Hospital, Freiburg, Germany

^3^Department of Intensive Care, Sleep Medicine and Mechanical Ventilation, Asklepios Fachkliniken Munich-Gauting, Germany

**ONLINE DATA SUPPLEMENT**

**Table S1.** Demographics, disease categories and co-morbidities

| **N= 25** | |
| --- | --- |
| Females, n (%) | 10 (40%) |
| Age, years | 64 (20;82) |
| BMI, kg/m² | 25.8 ± 7.7 |
| Pulmonary diseases, n (%)  COPD alone  COPD + OHS  COPD + destroyed lung  Neuromuscular disorders, n (%)  Amyotrophic lateral sclerosis  Spinal cord injury  Myopathy* | 14 (56%)  10 (40%)  3 (12%)  1 (4%)  11 (44%)  3 (12%)  4 (16%)  4 (16%) |
| Co-morbidities, n (%)  Arterial hypertension  Atrial fibrillation  Chronic heart failure  Mental diseases**  Chronic renal failure  Diabetes mellitus | 11 (44%)  4 (16%)  5 (20%)  18 (72%)  5 (20%)  4 (16%) |

*Multiminicore myopathy, mitochondrial myopathy, Curschmann-Steinert myotonic dystrophy and Duchenne muscular dystrophy

**Depression and/or anxiety and/or chronic pain syndrome

BMI: Body mass index; COPD: Chronic obstructive pulmonary disease; OHS: Obesity hypoventilation syndrome; NIV: Non-invasive ventilation; HMV: Home mechanical ventilation

Data are presented as mean ± standard deviation or as absolute numbers with the relative percentages in parentheses [n (%)]. For non-normally distributed data, median values with minimum and maximum ranges are given.

**Table S2.** Marital status and education

| N=25 | |
| --- | --- |
| Marital status, n (%)  Unmarried  Married  Divorced  Widowed  School-leaving qualification, n (%)  Secondary/High school graduate  Primary school  Non  University degree, n (%)  Completed apprenticeship, n (%)  Illiterate, n (%) | 7 (28%)  10 (40%)  4 (16%)  4 (16%)  7 (28%)  15 (60%)  3 (12%)  2 (8%)  14 (56%)  2 (8%) |

Data are presented as absolute number and perce

**Table S3.** Proportion of patients dissatisfied with specific aspects of daily life: NMD versus COPD.

| **Dissatisfied with** | **NMD (N=11)**  **%** | **COPD (N=14)**  **%** |
| --- | --- | --- |
| 1. Mobility | 85.7 | 45.5 |
| 2. Technical aids for communication | 78.6 | 45.5 |
| 3. Ability to communicate | 71.4 | 45.5 |
| 4. Life in general | 57.1 | 36.4 |
| 5. Social contacts | 57.1 | 36.4 |
| 6. Care dependency | 42.9 | 54.5 |
| 7. Religious life (N=17) | 44.4 | 37.5 |
| 8. Speech therapy (N=13) | 57.1 | 16.7 |
| 9. Medical care: outpatient | 35.7 | 36.4 |
| 10. No regrets about tracheostomy and invasive HMV | 42.9 | 18.2 |
| 11. Daily routine | 35.7 | 27.3 |
| 12. Mechanical ventilation | 42.9 | 18.2 |
| 13. Religious faith (N=11) | 22.2 | 37.5 |
| 14. Technical aids for mobilization | 28.6 | 27.3 |
| 15. Medical care: in-patient | 14.3 | 45.5 |
| 16. Occupational therapy (N=11) | 50.0 | 14.3 |
| 17. Nursing care | 14.3 | 36.4 |
| 18. Living situation | 14.3 | 27.3 |
| 19. Method of food intake | 7.1 | 27.3 |
| 20. Legal guardianship | 14.3 | 0 |
| 21. Physiotherapy | 14.3 | 0 |
| 22. Suction equipment | 7.1 | 9.1 |
| 23. Technical aids for personal hygiene | 0 | 9.1 |

NMD: neuromuscular disorders; HMV: home mechanical ventilation; COPD: Chronic Obstructive Pulmonary Disorders

Data are presented as percentage (%).

**Table S4.** Information on living conditions (N=25)

| 1. Mobility | |
| --- | --- |
| **Action**    Getting out of bed  Leaving the house  Excursions  Traveling | **Patient can perform action:**  **n = alone / with technical aids / with technical aids and personal help / not at all (%)**  n = 1 / 3 / 20 / 1 (4 %/ 12% / 80% /4%)  n = 0 / 1 / 15 / 9 (0% / 4% / 60% / 36%)  n = 0 / 0 / 13 / 12 (0% / 0% / 52% / 48%)  n = 0 / 0 / 2 / 23 (0% / 0% / 8% / 92%) |
| Examples of Excursions   | Accompanying spouse to work (butcher working at different farms)  Woodland walks, long walks along the river Rhein  Exploring the local area by car  Visiting family/relatives/friends at their homes  Attending a relative's wedding  Concerts, Cinema, Soccer game, Casino  Attending club meetings  Going to the Zoo, Bundesgartenschau (National Garden Show)  Street Festivals, Christmas markets  Going out for ice-cream, coffee, meals  Shopping tours  Visiting the Doctor  Examples of Traveling | | --- |   Family trip to Ostfriesland (northern Germany)  Vacation in Bavaria (with nursing service)  Visiting relatives at the Lake of Constance (with nursing service) | |

| 2. Technical aids for communication | |
| --- | --- |
| **Use of technical aids for communication n (%)**  Speaking valve  Mechanical ventilation, n (%)  Spontaneous breathing, n (%)  Speech computer, n (%)  Bell-system, n (%)  Other technical aids*, n (%)  *Reading: Book support, headrest, glasses, magnifying glass  Writing by hand: Pencil with hand-piece, alphabet board  Writing by computer: Eye-control, touchscreen, tablet  Hearing: Hearing device | 21 (84%)  5 (20%)  10 (40%)  6 (24%)  5 (20%)  8 (32%) |
| 3. Ability to communicate | |
| Reading, n (%)  Writing by hand, n (%)  Writing by computer, n (%)  Texting via phone, n (%)  Hearing, n (%)  Speaking, n (%)  Non-verbal communication, n (%) | 22 (88%)  19 (76%)  13 (52%)  11 (44%)  24 (96%)  13 (52%)  25 (100%) |
| 4. Life in general | |
| No further information specified. | |

| 5. Social contacts | |
| --- | --- |
| Number of patients with family members, n (%):  -with whom they are in regular contact  -with whom they live  Visits  Frequency (n=21), per week  Telecommunication  Frequency (n=9), per week  Loss of contact to close family members since invasive HMV establishment, n (%)  Number of patients with established relationships (couple), n (%)  Number of patients with friends, n (%)  -with whom they live  Visits  Frequency (N=14), per week  Telecommunication  Frequency (N=9), per week  Loss of contact with close friends since establishment of invasive HMV, n (%)  Social activities*, n (%)  *music society, sports club, choral society, self-help group, social associations, church community, face book | 23 (92%)  23 (92%)  15 (60%)  21 (84%)  3 (0.02;7)  9 (36%)  7 (1; 8)  6 (24%)  14 (56%)  15 (60%)  1 (4%)  14 (56%)  0.75 (0.25;7)  9 (36%)  1 (0.3;14)  14 (56%)  6 (24%) |

| 6. Care dependency | |
| --- | --- |
| Dependency on nursing care, n (%):  Feeding  Transfer  Grooming  Use of toilet  Bathing  Mobility (on level surface)  Stairs  Dressing  Barthel index*  *Barthel Index for Activities of Daily Living: range 0–100, where lower values indicate higher disability  Urinal catheter, n (%)  Continence: n (%)  Bladder  Bowel | 11 (44%)  20 (80%)  19 (76%)  23 (92%)  25 (100%)  21 (84%)  25 (100%)  24 (96%)  38.6 ± 22.1  8 (32%)  10 (40%)  17 (68%) |
| 7. Religious life | |
| Religion, n (%):  Catholic  Protestant  Muslim  Hindu  Belief in God/ higher power? n (%) | 21 (84%)  13 (52%)  5 (20%)  2 (8%)  1 (4%)  17 (68%) |

| 8. Speech therapy | |
| --- | --- |
| Speech therapy: n (%)  Session frequency per week (n=8),  Duration of session (n=8), min | 8 (32%)  1.8 ± 0.5  33.8 ± 6.9 |
| 9. Medical care: outpatient | |
| Frequency of outpatient consultations, per quarterly  Home visits by, n (%):  General practitioner  Respiratory physician  Other specialist physician*  Visit to doctor’s clinic**  * Neurology [N=6], Urology [N=3], Otolaryngology [N=2], Anesthesia [N=2], Psychology, Palliative medicine, Ophthalmology, Dentistry  ** General practitioner [N=2], Urology [N=2], Anesthesia, Surgery | 4 (1;18)  23 (92%)  4 (16%)  9 (36%)  4 (16%) |
| 10. No regrets about tracheostomy and invasive HMV | |
| NIV before invasive HMV:  Duration of NIV before invasive HMV (n= 7), months  Duration of invasive HMV, months  Surgical tracheotomy, n (%)  Time since tracheotomy, months  Unplanned hospital admissions following invasive HMV establishment, n (%) | 7 (28%)  20 (3;72)  19.9 (2.8;143.5)  17 (68%)  23 (6.2; 145.5)  18 (72%) |

| 11. Daily routine | |
| --- | --- |
| Sleep, h per day  Television, h per day  Social contacts, h per day  Nothing/idle, h per day  Personal hygiene/Nutrition, h per day  Education/Reading, h per day  Hobby, h per day  Telecommunication, h per day  Work, h per day | 9.7 ± 2.6  8.0 (0;14)  1.5 (0;14)  1.0 (0;10)  2.0 (1;4)  0.5 (0;10)  0.16 (0;6)  0.16 (0;10)  0 (0;5) |
| 12. Mechanical ventilation | |
| Settings:  Mode, n (%)  aPCV  PSV  PSIMV  IPAP, cmH_2_O  EPAP, cmH_2_O  Respiratory frequency (aPCV, N=15), per minute  LTOT, n (%)  Supplemental O2 (N=19), l/min  Continuous ventilation (24 hours per day), n (%)  Spontaneous Breathing (N= 14), hours per day | 15 (60%)  9 (36%)  1 (4%)  23.1 ± 8.7  5 (0;8)  16.1 ± 2.8  19 (76%)  2 (1;4)  11 (44%)  8.5 ± 5.7 |
| Equipment:  Second ventilator is available for emergencies, n (%)  Single/Double tube system, n (%)  Active/Passive exhalation valve, n (%)  Humidifier (active/passive/both), n (%)  Pulse oximeter, n (%)  Cuff-manometer, n (%)  Anesthesia bag, n (%)  Additional extern battery, n (%)  Long-term oxygen devices n (%)  Concentrator  Liquid oxygen tank  Compressed air connection  Mobile device  Tracheal cannula  Tracheal cannula diameter, mm  Substitute cannula, n (%)  Smaller cannula for emergency, n (%)  Diameter of emergency cannula, mm  Inhalation therapy, n (%)  During spontaneous breathing  During mechanical ventilation  Inhalation devices, n (%)  - Metered-dose inhaler + spacer  - Jet nebulizer: electric device/ compressed air connection  - Ultrasound nebulizer | 21 (84%)  123/2 (92/8%)  24/1 (96/4%)  11/11/3 (44/44/12%)  25 (100%)  24 (96%)  25 (100%)  14 (56%)  19 (76%)  8 (32%)  11 (44%)  3 (12%)  17 (68%)  10 (7;11)  25 (100%)  21 (84%)  8.5 (6;10)  21 (84%)  5 (20%)  18 (72%)  20 (80%)  1 (4%)  3/2 (12%/8%)  15 (60%) |
| 13. Role of religious faith in coping with disease | |
| No further information specified. | |

| 14. Technical aids for mobilization | |
| --- | --- |
| Use of technical aids for mobilization, n (%)  Wheelchair, electrical  Wheelchair, mechanical  Rollator  Lifter  Other technical aids*  * Ramp [3], headrest [2], walking aid | 25 (100%)  8 (32%)  20 (80%)  11 (44%)  9 (36%)  5 (20%) |
| 15. Medical care: inpatient | |
| Connected to a ventilation center  Distance to ventilation center, km  Patient knows how to reach ventilation center, n (%)  Target date for routine check-up, n (%)  Connected to other hospitals with focus on specific issues beyond respiration*, n (%)  * Urology, Cardiology, Orthopedics, Neurology, Dentistry, Hospital close to patient's home (2) | 25 (100%)  15 (0.1;104)  25 (100%)  19 (76%)  5 (20%) |
| 16. Occupational therapy | |
| Occupational therapy, n (%)  Session frequency per week, (N=8)  Duration of session (N=8), min | 8 (32%)  2.3 ± 1.3  35.0 ± 2.3 |
| 17. Nursing care | |
| Outpatient respiratory nursing care, n (%)  Hours per week  Nursing staff members, n (%)  Patients with family members involved in nursing care, n (%)  Spouse [wife/husband]  Parents [mother/father]  Children [daughter/son]  Siblings [sister/brother]  Patients with family members involved in respiratory care, n (%)  Patients with family members involved in basic care, n (%)  Suction frequency, per day  Secretion suctioning by, n (%)  Nursing service  Family members  Patient him/herself | 23 (92%)  168 (0;168)  9 (1;30)  12 (48%)  4 (16%) [3/1]  4 (16%) [4/3]  4 (16%) [3/1]  3 (12%) [3/0]  7 (28%)  12 (48%)  11.1 ± 5.4  24 (96%)  7 (28%)  3 (12%) |
| Nursing care insurance*, n (%)  Level 1  Level 2  Level 3  * Nursing care level according to the German nursing care insurance legislation (1 = lowest; 3 = highest). Level 3 indicates extreme dependency on nursing care, with the need for assistance with basic care, day and night. | 4 (16%)  8 (32%)  13 (52%) |

| 18. Living situation | |
| --- | --- |
| Large city (>100’000), n (%)  Mid-size city (>20’000), n (%)  Urban/small town (<20’000), n (%) | 15 (60%)  4 (16%)  6 (24%) |
| Barrier-free, n (%)  Stairs not barrier-free (N=7)  Access to garden/terrace/balcony, n (%) | 18 (72%)  4 (1;20)  20 (80%) |
| Private home, n= 18 (72%)  Live with family members, n (%)  Number of rooms  Size of apartment/house, m²  Size of main room, m²  Nursing facility, n= 7 (28%)  Living with family members, n (%)  Shared accommodation, n (%)  Nursing home, n (%)  Number of housemates in nursing facility  Size of main room, m² | 14 (56%)  3 (2;7)  87 (55;160)  20 (15;60)  1 (4%)  2 (8%)  5 (20%)  30 (7;32)  23.7 ± 6.0 |
| 19. Method of food intake | |
| Oral nutrition, n (%)  Enteral feeding (PEG tube), n (%)  Both, n (%) | 16 (64%)  3 (12%)  6 (24%) |
| 20. Legal guardianship | |
| Legal guardian/warrant, n (%)  Family member serving as legal guardian, n (%) | 18 (72%)  14 (56%) |

| 21. Physiotherapy |  |
| --- | --- |
| Physiotherapy, n (%)  Session frequency per week  Duration of session, min | 25 (100%)  2.2 ± 1.0  38.0 ± 10.9 |
| 22. Suction equipment | |
| Suction device, n (%)  Replacement device for suctioning, n (%)  Cough-assist, n (%) | 25 (100%)  22 (88%)  3 (12%) |
| 23. Technical aids for personal hygiene | |
| Use of technical aids for personal hygiene, n (%)  Health care bed  Commode chair  Other technical aids *  *bedhead hoist, toilet seat raiser, bedpan, urine bottle, disabled shower or bathtub, tub for washing hair in bed, stretcher or chair for shower | 24 (96%)  23 (92%)  15 (60%)  10 (40%) |

aPCV: assisted pressure-controlled ventilation; EPAP: Expiratory Positive Airway Pressure; HMV: Home mechanical ventilation; IPAP: Inspiratory Positive Airway Pressure; LTOT: long-term oxygen therapy; NIV: non-invasive ventilation; PEG: Percutaneous endoscopic gastrostomy; PSIMV: Pressure-Synchronized Intermittent Mandatory Ventilation; PSV: pressure-support ventilation; SRI: Severe Respiratory Insufficiency Questionnaire

Data are presented as absolute number and percentage [n (%)] or as mean ± standard deviation. For non-normally distributed data, median values with minimum and maximum ranges are given.

**Figure S1.** Daily routine of patients receiving invasive home mechanical ventilation


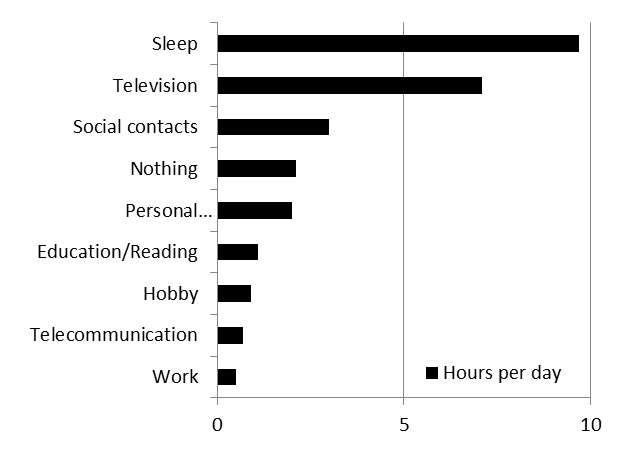

Supplement: Supplementary file 1 — Additional file 1. Table S1: Demographics, disease categories and co-morbidities. Table S2: Marital status and education. Table S3: Proportion of patients dissatisfied with specific aspects of daily life: NMD versus COPD. Table S4: Information on living conditions (N = 25). Figure S1: Daily routine of patients receiving invasive home mechanical ventilation. [file 13613_2018_384_MOESM1_ESM.doc]
